# Dicyanidobis(thio­urea-κ*S*)cadmium(II) monohydrate

**DOI:** 10.1107/S1600536810028710

**Published:** 2010-07-24

**Authors:** Mohammed Fettouhi, Muhammad Riaz Malik, Saqib Ali, Anvarhusein A. Isab, Saeed Ahmad

**Affiliations:** a Department of Chemistry, King Fahd University of Petroleum and Minerals, Dhahran 31261, Saudi Arabia; b Department of Chemistry, Quaid-i-Azam University, Islamabad, Pakistan; c Department of Chemistry, University of Engineering and Technology, Lahore 54890, Pakistan

## Abstract

In the title compound, [Cd(CN)_2_(CH_4_N_2_S)_2_]·H_2_O, the Cd atom lies on a twofold rotation axis and is bonded to two S atoms of thio­urea and two C atoms of the cyanide anions in a distorted tetra­hedral environment. The crystal structure is stabilized by N—H⋯N(CN), N—H⋯O, O—H⋯N and N—H⋯S hydrogen bonds.

## Related literature

For background to cadmium(II) complexes of thio­urea-type ligands, see: Corao & Baggio (1969 ▶); Malik *et al.* (2010 ▶); Marcos *et al.* (1998 ▶); Nawaz *et al.* (2010*a* ▶,*b* ▶); Wang *et al.* (2002 ▶). For the non-linear optical properties and semi-conducting applications of Cd–thio­urea complexes, see: Rajesh *et al.* (2004 ▶); Stoev & Ruseva (1994 ▶). For the structures of cyanido complexes of *d* ^10^ metal ions, see: Ahmad *et al.* (2009 ▶); Hanif *et al.* (2007 ▶); Yoshikawa *et al.* (2003 ▶).
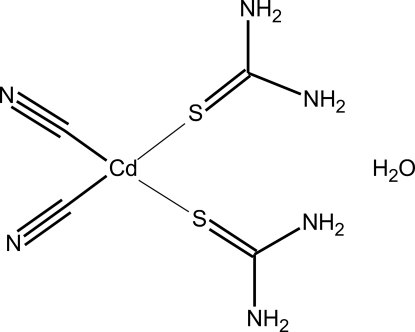


## Experimental

### 

#### Crystal data

[Cd(CN)_2_(CH_4_N_2_S)_2_]·H_2_O
*M* *_r_* = 334.70
Monoclinic, 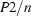

*a* = 10.5955 (6) Å
*b* = 4.0782 (3) Å
*c* = 13.4127 (8) Å
β = 98.738 (1)°
*V* = 572.84 (6) Å^3^
*Z* = 2
Mo *K*α radiation
μ = 2.25 mm^−1^
*T* = 294 K
0.29 × 0.28 × 0.24 mm

#### Data collection

Bruker SMART APEX area-detector diffractometer
Absorption correction: multi-scan (*SADABS*; Sheldrick, 1996 ▶) *T*_min_ = 0.561, *T*_max_ = 0.614
7211 measured reflections
1430 independent reflections
1376 reflections with *I* > 2σ(*I*)
*R*_int_ = 0.017

#### Refinement

*R*[*F* ^2^ > 2σ(*F* ^2^)] = 0.018
*wR*(*F* ^2^) = 0.043
*S* = 1.10
1430 reflections
86 parameters
All H-atom parameters refined
Δρ_max_ = 0.73 e Å^−3^
Δρ_min_ = −0.74 e Å^−3^

### 

Data collection: *SMART* (Bruker, 2008 ▶); cell refinement: *SAINT* (Bruker, 2008 ▶); data reduction: *SAINT*; program(s) used to solve structure: *SHELXS97* (Sheldrick, 2008 ▶); program(s) used to refine structure: *SHELXL97* (Sheldrick, 2008 ▶); molecular graphics: *ORTEP-3* (Farrugia, 1997 ▶); software used to prepare material for publication: *SHELXTL* (Sheldrick, 2008 ▶).

## Supplementary Material

Crystal structure: contains datablocks I, global. DOI: 10.1107/S1600536810028710/zl2290sup1.cif

Structure factors: contains datablocks I. DOI: 10.1107/S1600536810028710/zl2290Isup2.hkl

Additional supplementary materials:  crystallographic information; 3D view; checkCIF report

## Figures and Tables

**Table 1:** Hydrogen-bond geometry (Å, °)

*D*—H⋯*A*	*D*—H	H⋯*A*	*D*⋯*A*	*D*—H⋯*A*
N3—H5⋯O1^i^	0.72 (4)	2.26 (4)	2.961 (2)	166 (3)
N3—H4⋯S1^i^	0.90 (4)	2.61 (4)	3.470 (2)	159 (3)
N2—H3⋯N1^ii^	0.84 (3)	2.22 (3)	3.035 (2)	163 (3)
N2—H2⋯N1^iii^	0.78 (3)	2.51 (3)	3.286 (3)	171 (3)
O1—H1⋯N1^iv^	0.83 (3)	2.16 (3)	2.988 (2)	176 (3)

Symmetry codes: (i) 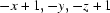; (ii) 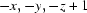; (iii) 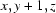; (iv) 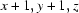.

**Fig. 1.**
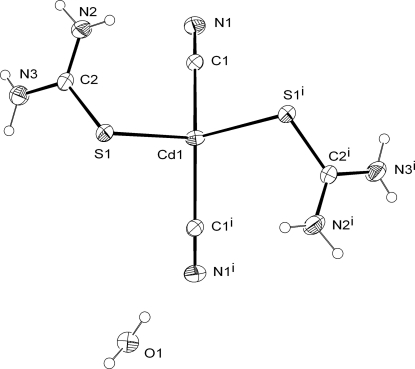
The molecular structure of the title compound with the atomic numbering scheme. Displacement ellipsoids are drawn at the 30% probability level (Symmetry code: i = 0.5-x, y, 0.5-z).

**Fig. 2.**
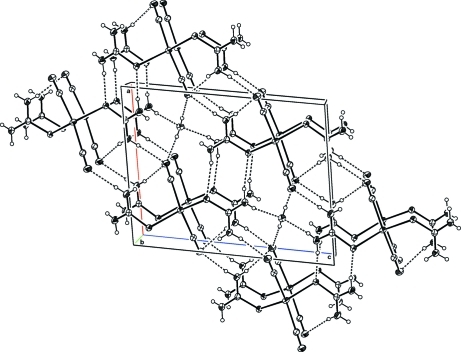
Packing diagram of the title complex showing the H-bonding interactions.

## supplementary crystallographic information

### Comment

The interest in cadmium(II) complexes of thiourea (Tu) arises because some of them exhibit non-linear optical properties (Rajesh *et al.*, 2004) and they are useful for the convenient preparation of cadmium sulfide based semiconducting materials by their thermal decomposition in air (Stoev *et al.*, 1994). Several crystallographic reports about cadmium(II) complexes of thiourea reveal that it coordinates to cadmium(II) *via* the sulfur atom (Corao *et al.*, 1969; Marcos *et al.*, 1998; Wang *et al.*, 2002). Recently, we have reported the crystal structures of cadmium(II) complexes of *N*,*N*'-dimethylthiourea (Dmtu), [Cd(Dmtu)_2_Cl_2_] (Malik *et al.*, 2010) and tetramethylthiourea (Tmtu), [Cd(Tmtu)_2_Br_2_] (Nawaz *et al.*, 2010*a*) and [Cd(Tmtu)_2_I_2_] (Nawaz *et al.*, 2010*b*). Herein, we report the crystal structure of a cadmium cyanide complex of thiourea, biscyanidobis(thiourea-kS)cadmium(II) monohydrate, [Cd(Tu)_2_(CN)_2_].H_2_O. The present investigation was carried out in view of our continuous interest in the structural chemistry of cyanido complexes of d^10^ metal ions with thiourea type ligands (Ahmad *et al.*, 2009; Hanif *et al.*, 2007).

In the title compound, the Cd atom is situated on a twofold axis of symmetry and is bonded to two cyanide carbon atoms and two sulfur atoms of thiourea (Figure 1). The four coordinate metal ion adopts a severely distorted tetrahedral geometry, the bond angles being in the range of 95.76 (4) - 143.5 (1) °. The Cd—S and Cd—C bond lengths are 2.6363 (5) Å and 2.211 (2) Å respectively. These are in agreement with those reported for related compounds (Marcos *et al.*, 1998; Malik *et al.* 2010; Nawaz *et al.*, 2010*a*,*b*; Wang *et al.*, 2002; Yoshikawa *et al.*, 2003). The two C—N bond lengths in thiourea, C2—N2 and C2—N3, are 1.312 (2) Å and 1.305 (2) Å respectively. The CNH_2_ fragments of the two thiourea molecules are essentially planar, the maximum deviation from the mean plane being for the nitrogen atoms with 0.03 (1) Å. These values are consistent with a significant CN double bond character and electron delocalization in the SCN_2_ moiety. To the best of our knowledge, this is the first X-ray structure of a cadmium complex having both sulfur containing ligands and cyanide in its coordination sphere.

The molecules pack to form columns parallel to the b direction (Figure 2). Within these columns, each metal ion interacts with two sulfur atoms of a neighboring molecule (Cd···S: 3.3140 (5) Å), hence extending the tetra-coordinate inner-sphere to a hexa-coordinate outer-sphere with a distorted octahedral environment. These interactions confer to the molecular columns a polymeric chain character.

Intermolecular hydrogen bonding takes place through N—H···S as well as N—H···N(CN) interactions (Table 1). The complex molecules also interact with the water molecules through C—N···H—O and N—H···O bonds. In this scheme the water molecule is tetrahedrally hydrogen bonded to four complex molecules. This generates a three-dimensional hydrogen bonding network where the molecular chains are interconnected through hydrogen bonding either directly or through the water molecules.

### Experimental

To 0.17 g (1.0 mmol) cadmium(II) cyanide (prepared by the reaction of CdCl_2_.H_2_O and KCN in 1:2 molar ratio in water) suspended in 15 mL water was added 2 equivalents of thiourea in methanol. Yellow precipitates formed, were filtered and the filtrate was kept for crystallization. Crystals were grown by slow evaporation of a water/methanol solution at room temperature.

### Refinement

All non-H atoms were refined anisotropically. Hydrogen atoms were located in a difference Fourier map and freely refined isotropically.

### Crystal data

**Table:**

[Cd(CN)_2_(CH_4_N_2_S)_2_]·H_2_O	*F*(000) = 328
*M**_r_* = 334.70	*D*_x_ = 1.940 Mg m^−^^3^
Monoclinic, *P*2/*n*	Mo *K*α radiation, λ = 0.71073 Å
Hall symbol: -P 2yac	Cell parameters from 7211 reflections
*a* = 10.5955 (6) Å	θ = 2.3–28.3°
*b* = 4.0782 (3) Å	µ = 2.25 mm^−^^1^
*c* = 13.4127 (8) Å	*T* = 294 K
β = 98.738 (1)°	Parallelepiped, yellow
*V* = 572.84 (6) Å^3^	0.29 × 0.28 × 0.24 mm
*Z* = 2	

### Data collection

**Table:**

Bruker SMART APEX area-detector diffractometer	1430 independent reflections
Radiation source: normal-focus sealed tube	1376 reflections with *I* > 2σ(*I*)
graphite	*R*_int_ = 0.017
ω scans	θ_max_ = 28.3°, θ_min_ = 2.3°
Absorption correction: multi-scan (*SADABS*; Sheldrick, 1996)	*h* = −14→14
*T*_min_ = 0.561, *T*_max_ = 0.614	*k* = −5→5
7211 measured reflections	*l* = −17→17

### Refinement

**Table:**

Refinement on *F*^2^	Secondary atom site location: difference Fourier map
Least-squares matrix: full	Hydrogen site location: inferred from neighbouring sites
*R*[*F*^2^ > 2σ(*F*^2^)] = 0.018	All H-atom parameters refined
*wR*(*F*^2^) = 0.043	*w* = 1/[σ^2^(*F*_o_^2^) + (0.0181*P*)^2^ + 0.3434*P*] where *P* = (*F*_o_^2^ + 2*F*_c_^2^)/3
*S* = 1.10	(Δ/σ)_max_ < 0.001
1430 reflections	Δρ_max_ = 0.73 e Å^−^^3^
86 parameters	Δρ_min_ = −0.73 e Å^−^^3^
0 restraints	Extinction correction: *SHELXL97* (Sheldrick, 2008), Fc^*^=kFc[1+0.001xFc^2^λ^3^/sin(2θ)]^-1/4^
Primary atom site location: structure-invariant direct methods	Extinction coefficient: 0.072 (2)

### Special details

**Table:**

Geometry. All e.s.d.'s (except the e.s.d. in the dihedral angle between two l.s. planes) are estimated using the full covariance matrix. The cell e.s.d.'s are taken into account individually in the estimation of e.s.d.'s in distances, angles and torsion angles; correlations between e.s.d.'s in cell parameters are only used when they are defined by crystal symmetry. An approximate (isotropic) treatment of cell e.s.d.'s is used for estimating e.s.d.'s involving l.s. planes.
Refinement. Refinement of *F*^2^ against ALL reflections. The weighted *R*-factor *wR* and goodness of fit *S* are based on *F*^2^, conventional *R*-factors *R* are based on *F*, with *F* set to zero for negative *F*^2^. The threshold expression of *F*^2^ > σ(*F*^2^) is used only for calculating *R*-factors(gt) *etc*. and is not relevant to the choice of reflections for refinement. *R*-factors based on *F*^2^ are statistically about twice as large as those based on *F*, and *R*- factors based on ALL data will be even larger.

### Fractional atomic coordinates and isotropic or equivalent isotropic displacement parameters (Å^2^)

**Table:**

	*x*	*y*	*z*	*U*_iso_*/*U*_eq_	
Cd1	0.2500	−0.06581 (5)	0.2500	0.03756 (9)	
S1	0.35766 (4)	0.31297 (11)	0.39821 (3)	0.03220 (11)	
C1	0.06927 (16)	−0.2357 (5)	0.29604 (12)	0.0340 (3)	
C2	0.28176 (16)	0.2070 (4)	0.49904 (12)	0.0322 (3)	
N1	−0.02293 (17)	−0.3264 (6)	0.31974 (14)	0.0499 (4)	
N2	0.16014 (18)	0.2695 (6)	0.49815 (14)	0.0535 (5)	
N3	0.3466 (2)	0.0687 (6)	0.57851 (14)	0.0518 (5)	
O1	0.7500	0.2524 (6)	0.2500	0.0451 (5)	
H1	0.811 (3)	0.376 (7)	0.267 (2)	0.059 (8)*	
H2	0.121 (3)	0.355 (8)	0.451 (2)	0.068 (9)*	
H3	0.127 (3)	0.243 (7)	0.551 (2)	0.056 (7)*	
H4	0.429 (4)	0.021 (8)	0.578 (3)	0.078 (10)*	
H5	0.313 (3)	0.013 (7)	0.619 (3)	0.064 (9)*	

### Atomic displacement parameters (Å^2^)

**Table:**

	*U*^11^	*U*^22^	*U*^33^	*U*^12^	*U*^13^	*U*^23^
Cd1	0.02792 (11)	0.05064 (14)	0.03652 (12)	0.000	0.01265 (7)	0.000
S1	0.0315 (2)	0.0395 (2)	0.02651 (18)	−0.00063 (16)	0.00713 (14)	0.00102 (16)
C1	0.0321 (8)	0.0416 (9)	0.0291 (7)	0.0028 (7)	0.0072 (6)	0.0029 (7)
C2	0.0352 (8)	0.0371 (8)	0.0249 (7)	−0.0004 (7)	0.0063 (6)	−0.0031 (6)
N1	0.0367 (8)	0.0677 (12)	0.0477 (9)	−0.0040 (8)	0.0145 (7)	0.0077 (9)
N2	0.0403 (9)	0.0892 (16)	0.0338 (8)	0.0155 (10)	0.0148 (7)	0.0124 (9)
N3	0.0417 (9)	0.0832 (15)	0.0319 (8)	0.0100 (9)	0.0099 (7)	0.0164 (9)
O1	0.0373 (10)	0.0563 (13)	0.0423 (10)	0.000	0.0076 (8)	0.000

### Geometric parameters (Å, °)

**Table:**

Cd1—C1^i^	2.2108 (17)	C2—N2	1.312 (2)
Cd1—C1	2.2108 (17)	N2—H2	0.78 (3)
Cd1—S1	2.6363 (5)	N2—H3	0.84 (3)
Cd1—S1^i^	2.6363 (5)	N3—H4	0.90 (4)
S1—C2	1.7300 (17)	N3—H5	0.72 (4)
C1—N1	1.134 (2)	O1—H1	0.83 (3)
C2—N3	1.305 (2)		
			
C1^i^—Cd1—C1	143.47 (10)	N3—C2—S1	119.80 (15)
C1^i^—Cd1—S1	95.76 (4)	N2—C2—S1	121.13 (14)
C1—Cd1—S1	105.48 (5)	C2—N2—H2	120 (2)
C1^i^—Cd1—S1^i^	105.48 (5)	C2—N2—H3	120.4 (19)
C1—Cd1—S1^i^	95.76 (4)	H2—N2—H3	119 (3)
S1—Cd1—S1^i^	108.26 (2)	C2—N3—H4	119 (2)
C2—S1—Cd1	104.11 (6)	C2—N3—H5	119 (3)
N1—C1—Cd1	179.22 (18)	H4—N3—H5	122 (3)
N3—C2—N2	119.05 (18)		

Symmetry codes: (i) −*x*+1/2, *y*, −*z*+1/2.

### Hydrogen-bond geometry (Å, °)

**Table:**

*D*—H···*A*	*D*—H	H···*A*	*D*···*A*	*D*—H···*A*
N3—H5···O1^ii^	0.72 (4)	2.26 (4)	2.961 (2)	166 (3)
N3—H4···S1^ii^	0.90 (4)	2.61 (4)	3.470 (2)	159 (3)
N2—H3···N1^iii^	0.84 (3)	2.22 (3)	3.035 (2)	163 (3)
N2—H2···N1^iv^	0.78 (3)	2.51 (3)	3.286 (3)	171 (3)
O1—H1···N1^v^	0.83 (3)	2.16 (3)	2.988 (2)	176 (3)

Symmetry codes: (ii) −*x*+1, −*y*, −*z*+1; (iii) −*x*, −*y*, −*z*+1; (iv) *x*, *y*+1, *z*; (v) *x*+1, *y*+1, *z*.
